# Refractory Acquired Amegakaryocytic Thrombocytopenia with Rapid Progression to Aplastic Anaemia in SLE

**DOI:** 10.31138/mjr.110823.raa

**Published:** 2023-08-11

**Authors:** Bana Hadid, Arif Kodza, Sumatha Channapatna Suresh, Aleksander Feoktistov

**Affiliations:** 1 State University of New York Downstate Health Sciences University, College of Medicine, Brooklyn, NY, USA,; 2 State University of New York Downstate Health Sciences University, Department of Internal Medicine, Brooklyn, NY, USA,; 3 NYC Health and Hospitals/Kings County Hospital Centre, Department of Rheumatology, NY, USA

**Keywords:** acquired amegakaryocytic thrombocytopenia, AAMT, lupus thrombocytopenia, bone marrow megakaryocyte hypoplasia, refractory thrombocytopenia, early bone marrow transplant

## Abstract

Acquired amegakaryocytic thrombocytopenia (AAMT) is a rare cause of thrombocytopenia seen in systemic lupus erythematosus (SLE) that is frequently misdiagnosed as immune thrombocytopenic purpura (ITP). Often patients do not respond to standard ITP treatment. Prompt bone marrow biopsy and further workup should ensue as it is a diagnosis of exclusion. While no standard guidelines exist, the mainstay of treatment is immunosuppressive therapy. Some cases are refractory and should have a follow-up biopsy, typically showing worsening disease. The exact pathogenesis is unclear; multiple mechanisms may be involved, suggesting AAMT may be a syndrome of various aetiologies rather than a distinct pathology. A common complication is aplastic anaemia, and the patient may need a haematopoietic stem cell transplant (HSCT). We present a young man with severe refractory AAMT in the setting of SLE that progressed to aplastic anaemia and required an HSCT. We then discuss and interpret the literature on AAMT.

## INTRODUCTION

Thrombocytopenia is common in systemic lupus erythematosus (SLE). Immune thrombocytopenic purpura (ITP) is the most frequent aetiology and typically responds to corticosteroids and conventional immunosuppressants.^1^ More severe cases usually respond to intravenous immunoglobulin (IVIG), rituximab, and eltrombopag. Another aetiology, albeit rare, is acquired amegakaryocytic thrombocytopenia (AAMT). It presents similarly to ITP and is frequently misdiagnosed; hence, the incidence is often higher than reported.^2^ However, its pathogenesis differs, and bone marrow (BM) biopsy does not show a reactive increase of megakaryocytes.^3,4^ It is refractory to most immunosuppressants,^5^ and no standard treatment is available. The clinical course is highly variable and often progresses to aplastic anemia.^6^ Here, we present a case of AAMT in SLE that was refractory to multiple immunosuppressive therapies. Our patient developed aplastic anaemia with transfusion dependence and required a bone marrow transplant (BMT). Early diagnosis of AAMT is crucial when thrombocytopenia is not responsive to standard treatment as these patients may benefit from an early BMT.

## CASE DESCRIPTION

Written informed consent was obtained from the patient prior to completing this case report.

A 28-year-old man presented with two weeks of abrupt onset petechiae. He stated he had not been in contact with anyone who was symptomatic with an infectious illness, had no recent viral illness, was never sexually active, and was not on any medications. He denied haematuria, haematochezia, hair loss, joint pains, oral ulcers, Raynaud’s phenomena, dry eyes, and dry mouth. He had a family history of pernicious anaemia in one sister, pityriasis lichenoides et varioliformis acute in another, and rheumatoid arthritis in his maternal grandmother. Physical exam revealed petechiae on bilateral forearms but was otherwise unremarkable, with no hepatosplenomegaly or mucosal lesions.

Further workup showed severe thrombocytopenia (2.0x10^9^/L), anaemia (10^9^ g/L), and leukopenia (3.49 x10^9^/L). His autoimmune serologies showed positive antinuclear autoantibodies (ANA), double-stranded DNA autoantibodies (anti-dsDNA), Sjögren’s-syndrome-related antigen A/B autoantibodies (anti-SSA/SSB), and DNA extractable nuclear antigen ribonucleoprotein autoantibodies (anti-ENA-RNP). They were negative for anti-Smith autoantibodies (anti-Sm), beta-2-glycoprotein autoantibodies (anti-B2gP), and cardiolipin autoanti-bodies (anti-cardiolipin). He also had low complement 4 levels. Peripheral smear was negative for schistocytes. There were no peripheral smear or laboratory evidence of haemolysis. He also had a low reticulocyte count. Infectious workup (Ebstein Barr Virus [EBV], cytomegalovirus [CMV], Human Immunodeficiency Virus [HIV], and Hepatitis C Virus [HCV]) was negative. He tested positive for Hepatitis B core antibody, but that was attributed to the IVIG transfusions.^7^ Nevertheless, he was started on tenofovir to treat Hepatitis B Virus [HBV]. The working diagnosis at this point was ITP secondary to SLE. While ITP is defined as isolated thrombocytopenia, some patients with lupus can have thrombocytopenia as their only symptom before diagnosis.^8^ One study found that patients with an initial diagnosis of ITP who later developed SLE had significantly lower haemoglobin levels/anaemia compared to those who did not develop SLE.^9^ Furthermore, SLE is associated with various hemato-logic abnormalities.^1^ Our patient likely had undiagnosed SLE at the time of his presentation, accounting for his pancytopenia rather than isolated thrombocytopenia. Additionally, ITP is the most common aetiology of thrombocytopenia in SLE.^1^

**Figure 1.**
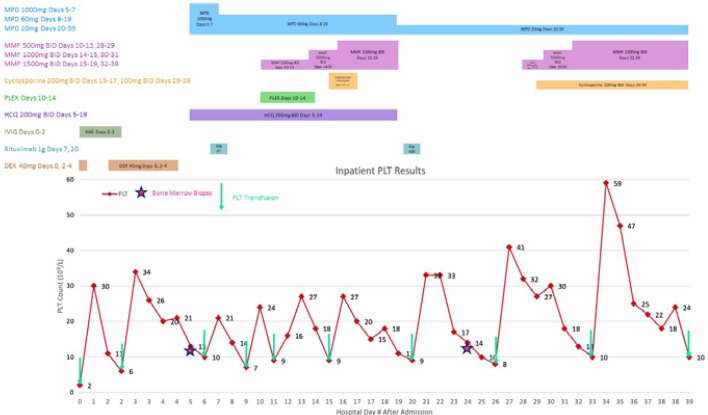
Inpatient clinical course and platelet (PLT) level results. MPD: methylprednisolone; MMF: mycophenolate mofetil; PLEX: plasma exchange; HCQ: hydroxychloroquine; IVIG: intravenous immunoglobulin; DEX: dexamethasone.

The patient was started oral steroids and IVIG. He was also given rescue platelet transfusions for critically low levels (<10 x10^9^/L). Due to lack of response, a BM biopsy was performed, which revealed hypocellular marrow (30-40% cellularity), no evidence of leukaemia or lymphoma, and severely decreased megakaryocytes. This was suggestive of AAMT. The patient never exhibited any signs of active bleeding. He was given plasma exchange, IV parenteral steroids, and other immunosuppressants, as summarised in **Figure 1**, but the patient remained thrombocytopenic (platelet count below 30x10^9^/L). Of note, cyclophosphamide was offered, but the patient refused due to its side effects. When the patient became leukopenic, we considered medication-induced pancytopenia and hydroxychloroquine, mycophenolate mofetil, cyclosporine, and tenofovir were held in addition to decreasing the methylprednisolone dose. However, the patient continued to be pancytopenic.

**Figure 2.**
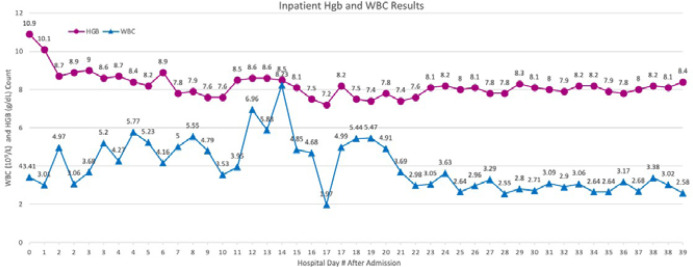
Inpatient haemoglobin (Hgb) and white blood cell (WBC) count results.

A second BM biopsy was performed as the pancytopenia did not improve, and the results showed worsening hypocellular marrow with cellularity to 10–20% with occasional histiocytes showing hemophagocytosis, concerning for hemophagocytic lymphohistiocytosis (HLH). However, the patient did not have clinical or laboratory features of HLH. Serum triglycerides, ferritin, fibrinogen, and serum interleukin two receptor (sIL-2R) were all within normal limits. The diagnosis of AAMT secondary to SLE was maintained. The complicated hospital course is summarised in **Figures 1** and **2**.

**Figure 3.**
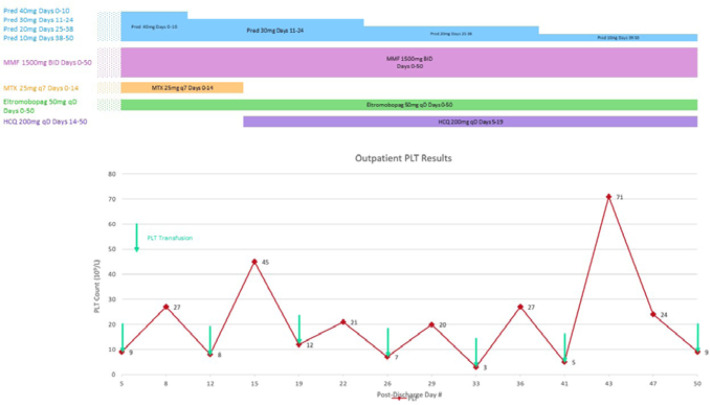
Outpatient clinical course and platelet (PLT) level results. Pred: Prednisolone; MMF: mycophenolate mofetil; MTX: methotrexate; HCQ: hydroxychloroquine.

**Figure 4.**
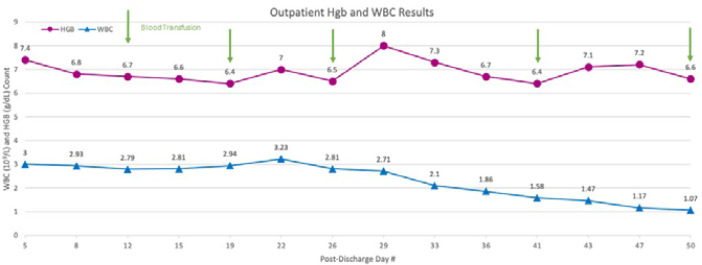
Outpatient haemoglobin (Hgb) and white blood cell (WBC) count results.

The patient was discharged, and we continued to follow him outpatient. Eltrombopag was added to his regimen. He still did not respond to immunosuppressant treatment. He remained platelet transfusion dependent, eventually became blood transfusion dependent due to worsening anaemia, and also developed worsening leukopenia. Still, he never displayed any signs of bleeding. The patient was ultimately referred for BMT for aplastic anaemia, which was diagnosed based on his laboratory blood analysis which confirmed low cell counts in all cell lines in addition to the hypocellularity seen on the second BM biopsy. The patient’s outpatient management course is summarized in **Figures 3** and **4**.

## DISCUSSION

Thrombocytopenia (platelet count <100x10^9^/L) can be found in 7–30% of SLE cases.^1^ Up to 14% of patients with lupus can have thrombocytopenia as the sole finding for up to 10 years before diagnosis.^8^ SLE thrombocytopenia is an independent mortality predictor and is associated with a worse prognosis.^1^ Among lupus thrombocytopenia cases, severe thrombocytopenia (platelet count <50x10^9^/L) can be seen in 27.9% of them as per a recent Chinese cohort study.^10^ That same study demonstrated severe thrombocytopenia to be associated with relatively quiet lupus and decreased survival rate.^10^ Our patient presented with isolated severe thrombocytopenia in the setting of asymptomatic, undiagnosed lupus that was refractory to most treatments.

Some of the most common causes of thrombocytopenia in lupus are medications, viral infections (EBV, CMV, HIV, HCV, parvovirus, HBV), antiphospholipid syndrome, and thrombotic microangiopathies,^1^ all of which were ruled out in our patient. Other mechanisms are impaired megakaryocyte production and immune-mediated platelet destruction.^1^ The latter is the most common aetiology,^1^ which prompted our patient, who had severe thrombocytopenia and positive SLE serologies with a negative workup, to be treated with steroids and IVIG. When his BM biopsy was done due to lack of response after five days, it showed depleted megakaryocytes, making ITP a very unlikely diagnosis. ITP is due to autoantibodies targeting antigenic glycoprotein on platelets which are then sequestered in the spleen and destroyed.^1^ BM biopsy in ITP typically shows normal or increased megakaryocytes,^1^ inconsistent with our patient.

Our patient’s initial biopsy was done after five days of no response to aggressive immunotherapy for presumed ITP. There is no standardised guideline regarding when to perform a BM biopsy after patients with thrombocytopenia do not respond to initial therapies. Case reports have described performing biopsies any time after three days to five months to possibly years.^2,11,12^ Therefore, diagnosis, and thus treatment, of AAMT can be delayed when a low platelet count is further investigated. Studies are needed to compare the timeframes of obtaining BM biopsies in patients presenting with grave thrombocytopenia. An additional point to consider is that megakaryocyte counts in the BM may have a predictive value in determining response to therapy in patients with SLE who have thrombocytopenia.^13^ When thrombocytopenia is persistent and severe, one should not hesitate to repeat a BM biopsy. Our patient displayed a worsening BM response, consistent with other published studies.^14–16^ In two of these studies, AAMT progressed to aplastic anemia.^15,16^ Therefore, repeat biopsy has the potential to predict clinical outcomes. Furthermore, HLH should be suspected with unremitting thrombocytopenia, but this was deemed unlikely in our case. Although some hemophagocytic histiocytes were noted on repeat BM biopsy, he did not fulfil HLH criteria due to the absence of fever, organomegaly, elevated ferritin, and elevated sIL-2R.

Our patient’s biopsy was consistent with AAMT.^5^ AAMT is a diagnosis of exclusion, and other causes of acquired thrombocytopenia should be ruled out. For example, endogenous stimuli can suppress megakaryocyte maturation due to antibody or T-cell-mediated immunity.^4,17^ AAMT has been associated with systemic lupus erythematous, eosinophilic fasciitis, systemic sclerosis, and adult-onset Still’s disease, among other autoimmune disorders.^18–20^ Our patient did not have clinical features of these disorders. Lastly, AAMT has been described as an early manifestation of a stem cell abnormality, such as a precursor to acute myeloid leukaemia, myelodysplastic syndrome, and non-Hodgkin’s lymphoma.^2^ However, our patient’s BM biopsies were negative for malignancy. In our case, we exhausted the various aetiologies of thrombocytopenia and found that he likely has AAMT secondary to SLE. While the exact pathogenesis remains unknown, autoantibodies against thrombopoietin (THPO) or THPO receptor are thought to be involved.^5,14,17,21^ The most common antibodies associated with lupus thrombocytopenia are anti-Glycoprotein IIa/IIIb and anti-THPO antibody.^4^ THPO binds to its receptor and stimulates megakaryocyte maturation and platelet production. Antibodies against this cytokine or its receptor inhibit megakaryopoiesis leading to low or absent megakaryocytes in the BM.^4,17^ Anti-THPO antibodies are associated with bone marrow megakaryocytic hypoplasia and poor response to standard immunosuppressants.^4,17^ On the other hand, anti-glycoprotein IIa/IIIB is associated with peripheral platelet destruction or splenic sequestration, hence associated with high megakaryocyte density compared to anti-THPO antibodies.

There are several case reports on AAMT but no extensive studies to investigate the clinical outcomes of this disease. There are also no randomized clinical trials (RCTs) for treatment of the disease. Immunosuppression, however, remains the initial foundation of therapy. The following medications and therapies have been used for the treatment of AAMT:^2,3,5,11,14,22,23^ steroids (which suppress autoimmunity mediated by B and T cells), IVIG (which binds antibodies against THPO and megakaryocytes), plasma exchange (which removes and filters antibodies from the patient’s plasma), rituximab (which suppresses autoantibody production by B cells), cyclosporine (which is a calcineurin inhibitor that suppresses T-cell activation), anti-thymocyte globulin (which suppresses autoimmunity mediated by T cells and directly stimulates haematopoiesis), cyclophosphamide (which is immunosuppressive), azathioprine and mycophenolate mofetil (which diminish immune cell proliferation, especially in patients with systemic lupus erythematosus), eltrombopag (which stimulates THPO receptor), and BM transplant (which replaces a patient’s BM, including their megakaryocytes). A variety of these drugs were tried on our patient. Still, he progressed to aplastic anaemia that was transfusion-dependent, a known complication of the intense autoimmune processes of AAMT.^6^

Before the 2000s, anti-thymocyte globulins (ATG) and cyclosporine were successfully used to treat AAMT.^24,25^ One of the most extensive case series on AAMT by Manoharan et al. has shown responses to various treatments, and patients treated with ATG had the best outcomes.^26^ Few other case reports have shown a failed response to ATG but a good response to cyclophosphamide or cyclosporin.^22,24,27^ Recent studies have demonstrated success with rituximab, eltrombopag, romiplostim, and BMT.^11,14,23,28^ Eltrombopag was approved for use in primary ITP, and recently a small RCT showed promising results with eltrombopag in ITP associated with connective tissue disorders.^29^ Nevertheless, there is a paucity in the literature on the use of any of these treatments in AAMT. A couple of case reports discuss the response with eltrombopag and romiplostim.^23,28^ Eltrombopag, specifically, stimulates THPO receptor at a transmembrane region of THPO receptor, while THPO binds to the distal portion, cytokine receptor homologous domain 2, of THPO to activate megakaryocyte maturation.^30^ This suggests that eltrombopag may provide an adequate response in the presence of anti-THPO receptor antibodies.^23^ Review of our patient’s case in combination with other cases in the literature suggests that AAMT may describe a syndrome secondary to various mechanisms rather than a discrete disease with a defined pathogenic process. Further research is needed to elucidate the pathogenesis and treatment of this disease.

The prognosis for AAMT varies from immediate remission after therapy to a long relapsing-remitting course to progression to aplastic anaemia in 1 month to 2 years.^6,15,21,22^ Our patient rapidly progressed to aplastic anaemia in a span of two weeks. However, once aplastic anaemia has occurred, the prognosis is poor. Patients may require a haematopoietic stem cell transplant (HSCT) to achieve remission.^11^ HSCT cases have shown increased survival in congenital amegakaryocytic thrombocytopenia.^31^ Early HSCT may also be beneficial in AAMT; to the best of our knowledge, two case reports show favourable outcomes with HSCT in patients with AAMT.^11,21^

## CONCLUSION

AAMT is one of the uncommon haematological manifestations of lupus. It is underrecognised and underdiagnosed as most thrombocytopenia in SLE is assumed to be ITP. Although ITP is the most common cause of thrombocytopenia in SLE, a suspicion of AAMT is essential, and prompt BM biopsy should be obtained, especially when there is no response to standard steroids and IVIG. Biopsy shows low or absent megakaryocytes. Refractory AAMT in a young patient should be treated aggressively, and repeat BM biopsy may sometimes be essential when there is no response to standard immunosuppression. Furthermore, early referral to HSCT may be the next logical step.
